# Forward dynamic simulation of Japanese macaque bipedal locomotion demonstrates better energetic economy in a virtualised plantigrade posture

**DOI:** 10.1038/s42003-021-01831-w

**Published:** 2021-03-08

**Authors:** Hideki Oku, Naohiko Ide, Naomichi Ogihara

**Affiliations:** 1 Department of Mechanical Engineering, Faculty of Science and Technology, Keio University, Yokohama, Japan; 2 Department of Biological Sciences, Graduate School of Science, The University of Tokyo, Tokyo, Japan

**Keywords:** Biological anthropology, Computational models

## Abstract

A plantigrade foot with a large robust calcaneus is regarded as a distinctive morphological feature of the human foot; it is presumably the result of adaptation for habitual bipedal locomotion. The foot of the Japanese macaque, on the other hand, does not have such a feature, which hampers it from making foot–ground contact at the heel during bipedal locomotion. Understanding how this morphological difference functionally affects the generation of bipedal locomotion is crucial for elucidating the evolution of human bipedalism. In this study, we constructed a forward dynamic simulation of bipedal locomotion in the Japanese macaque based on a neuromusculoskeletal model to evaluate how virtual manipulation of the foot structure from digitigrade to plantigrade affects the kinematics, dynamics, and energetics of bipedal locomotion in a nonhuman primate whose musculoskeletal anatomy is not adapted to bipedalism. The normal bipedal locomotion generated was in good agreement with that of actual Japanese macaques. If, as in human walking, the foot morphology was altered to allow heel contact, the vertical ground reaction force profile became double-peaked and the cost of transport decreased. These results suggest that evolutionary changes in the foot structure were important for the acquisition of human-like efficient bipedal locomotion.

## Abstract (web summary)

Oku, Ide and Ogihara use forward dynamic simulation to virtually manipulate the foot posture of the Japanese macaque from a digitigrade to plantigrade posture for bipedal locomotion. This postural shift results in greater energetic economy despite the macaque not having anatomy adapted for bipedal movement. Structural changes in the foot are likely a key component of the acquisition of human-like bipedal locomotion.

## Introduction

Nonhuman primates such as chimpanzees and macaques generally have the ability to walk bipedally. However, bipedal locomotion of an inherently quadrupedal primate is different from that observed in humans. For example, the hip and knee joints are more flexed throughout the gait cycle^1^. Furthermore, nonhuman primates do not generally exhibit the characteristic double-peaked vertical ground reaction force (GRF) profile seen in humans (see Schmitt^2^ for a review). Some chimpanzees are reportedly capable of generating double-peaked vertical GRF^3^, but the force profile is not the clear two-peak pattern of humans. Owing to these kinematic and kinetic differences, phase and/or amplitude of fluctuation of the body’s center of mass (COM) and hence mutual transfer of potential and kinetic energy during bipedal locomotion are somewhat different between humans and nonhuman primates, resulting in lower energy recovery from exchange of potential and kinetic energy during bipedal locomotion in nonhuman primates than in humans^4–7^.

**Fig. 1:**
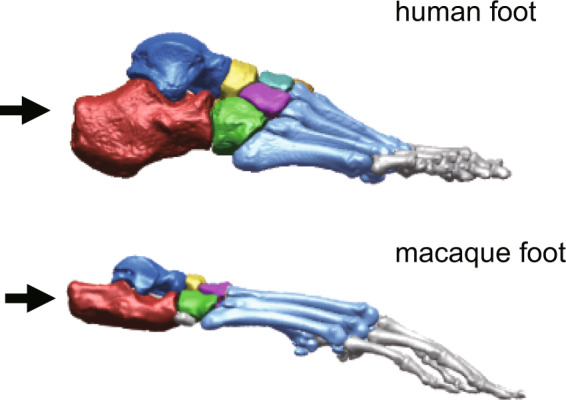
Human and macaque feet. The arrows indicate the calcaneus. The human foot possesses a longitudinal arch with an enlarged, robust calcaneus, the tuberosity of which points posteriorly and inferiorly, allowing prominent heel strike. The macaque foot does not possess such a longitudinal arch, and its calcaneal tuberosity points posteriorly and superiorly.

Such differences in bipedal walking between humans and nonhuman primates exist because of structural differences in the musculoskeletal system. Among these differences, those of the foot, along with the pelvis and femur, seem to be important, as it is the most distal segment of the body that directly interacts with the ground^8^. The structure of the human foot is different from those of nonhuman primates; this is presumably the result of morphological adaptation for the generation of stable and efficient bipedal locomotion^9–13^. For example, the opposability of the hallux, and hence, the prehensile capability of the foot in nonhuman primates, is absent in the human foot. Furthermore, the human foot uniquely possesses a longitudinal arch with an enlarged, robust calcaneus, the tuberosity of which points posteriorly and inferiorly, allowing prominent heel strike during plantigrade gait in humans^14–17^. However, the primate foot does not possess such a longitudinal arch, and its calcaneal tuberosity points posteriorly and superiorly (Fig. 1). Furthermore, the range of dorsiflexion at the ankle joint is too restricted to allow heel contact during walking^18,19^, possibly because the metatarsophalangeal (MP) joint must be dorsiflexed before touch down and hence the flexor digitorum longus is stretched at the time of foot contact^20^. Therefore, macaques do not walk bipedally with heel contact. African great apes have plantigrade feet with its calcaneal tuberosity directed posteriorly without being inclined substantially and hence can walk with a heel strike^15,21–24^, but the heel and midfoot often contact the ground simultaneously^24^, and the calcaneus is not as derived as that in humans. Therefore, a robust calcaneus is considered to be one of the fundamental morphological adaptations for the acquisition of habitual bipedal locomotion in humans.

Recently, it was hypothesized that bipedal walking with plantigrade foot and heel contact is energetically more economical than that with digitigrade foot and forefoot contact. For example, Cunningham et al.^25^ experimentally demonstrated that human walking with plantigrade feet required less energy than that with digitigrade feet because of the reduced energy loss during collision between the foot and the ground, the increased utilization of the inverted pendulum mechanism^26^, and a reduced ankle joint moment. Webber and Raichlen^27^ studied human walking with foot-ground contact with the balls of the foot initially with heel contact occurring later in the step and noted that the decrease in the energetic cost occurred in human walking with heel contact because of the increase in the effective limb length owing to the larger anterior translation of the center of pressure (COP). These findings suggested the possible functional significance of the evolution of the plantigrade foot to allow bipedal locomotion with heel contact, as observed in humans. However, in those studies, increased energy consumption with forefoot contact may have been observed, possibly because the human musculoskeletal system, in addition to the motor control system, has been adapted to heel but not to forefoot strike bipedal locomotion over the course of human evolution. How the evolutionary changes in the structure of the foot and the way it interacts with the ground affect the mechanics and energetics of bipedal locomotion in early hominins whose musculoskeletal structure differed from humans remains unclear.

Our group has been investigating bipedal locomotion in Japanese macaques as a way to achieve a better understanding of the evolution of human bipedalism^5,19,28–32^. Chimpanzees were commonly thought to be the most appropriate living proxies for the last common ancestor of both humans and chimpanzees. However, chimpanzees are well specialized to suspensory-inclined locomotion and knuckle-walking in terms of musculoskeletal anatomy^33–35^. After the discovery of *Ardipithecus*, it was hypothesized that, unlike modern apes, the last common ancestor was a relatively large-bodied but non-suspensory arboreal clamberer albeit whose trunk and aspects of their limbs were more derived than that in general pronograde quadrupeds, such as early and middle Miocene African apes^36^ [but see Pilbeam and Lieberman^37^ for alternative arguments]. Therefore, utilizing a generalized quadrupedal primate such as the macaque that is less-specialized in terms of locomotor anatomy^35^ as a living model of the hypothetical protohominids is of increasing importance to clarifying and reconstructing the evolution of bipedal locomotion in the early hominins.

If the feet of bipedal Japanese macaques could somehow be manipulated to that of humans to allow for bipedal gait, the possible changes that might occur in bipedal locomotion could be observed, which would offer an opportunity to investigate the evolutionary advantage of human foot morphology in hypothetical protohominids. However, we certainly cannot replace the macaque foot with a human foot in vivo to evaluate the effect of changes in foot morphology on the mechanics and energetics of bipedal locomotion. To investigate possible changes in bipedal locomotion resulting from alterations in the musculoskeletal system, an alternative method is necessary.

A forward dynamic musculoskeletal simulation is a computational technique used to calculate muscle-driven skeletal motions by integrating differential equations that define the dynamics of a musculoskeletal system. Many musculoskeletal forward dynamic simulations of bipedal walking have been conducted to clarify the biomechanics and motor control of human locomotion^38–57^. This predictive simulation technique has also been conducted in the field of physical anthropology to help understand the evolution of human bipedal locomotion^58–61^. A forward dynamic simulation of bipedal walking in the Japanese macaque could enable changes in the mechanics and energetics of bipedal walking resulting from virtual alteration of the musculoskeletal system to be predicted. Therefore, in the present study, we constructed a muscle-driven forward dynamic simulation of bipedal locomotion in the Japanese macaque based on a two-dimensional (2D) neuromusculoskeletal model and evaluated how virtual manipulation of foot morphology affects the kinematics, dynamics, and energetics of bipedal locomotion. Specifically, we investigated how the alteration of the foot from digitigrade to plantigrade by the inferior translation of the calcaneal tuberosity, which allows heel strike bipedal locomotion, affects the kinematics, dynamics, and energetics of bipedal locomotion in the Japanese macaque using computer simulation.

## Results

### Evaluation of the generated bipedal locomotion

**Fig. 2:**
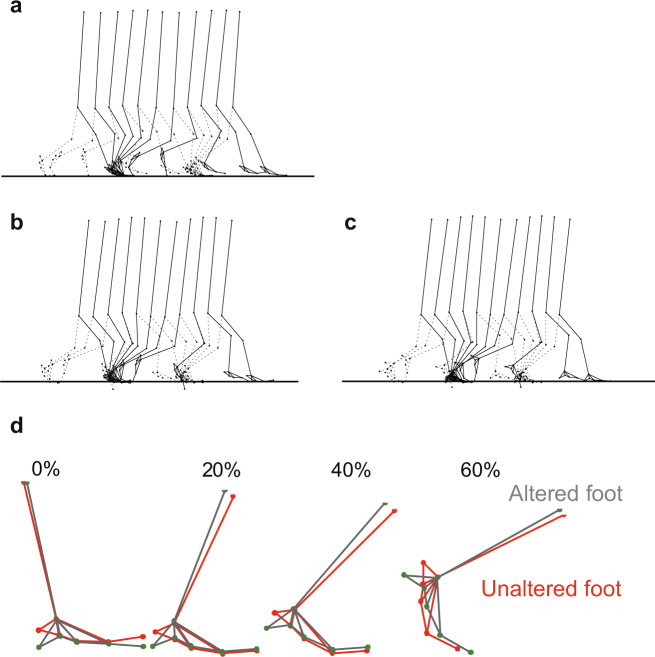
Stick diagrams of the measured locomotion of the Japanese macaque compared with the simulated locomotion with unaltered and altered foot morphology (traced every 10% of the gait cycle). **a** Measured locomotion. **b** Simulated locomotion with unaltered foot morphology. **c** Simulated locomotion with altered foot morphology. **d** Comparisons of the foot and shank movements during the stance phase of bipedal locomotion between before and after the alteration in foot morphology.

**Fig. 3:**
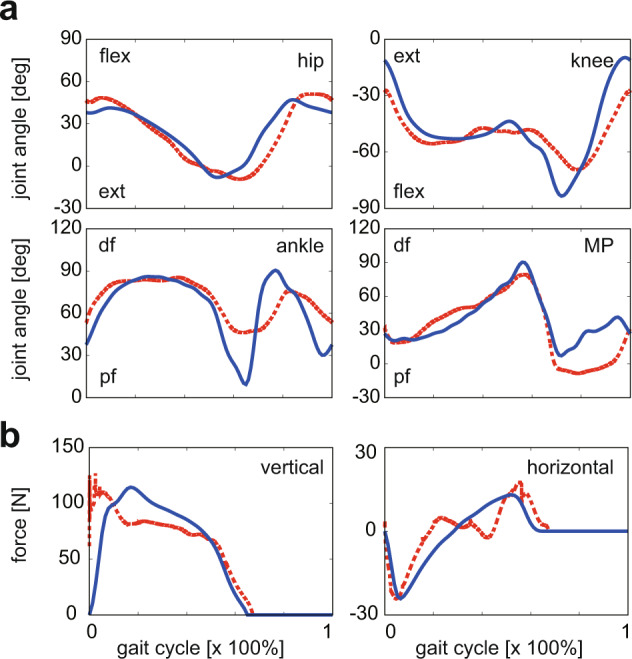
Comparisons between the simulated locomotion in the Japanese macaque model with the unaltered foot and the experimentally measured bipedal walking in the Japanese macaque. Changes in (**a**) joint angles and (**b**) GRFs over time are expressed as a percentage of the gait cycle. Blue solid line = actual. Red dotted line = simulated.

**Fig. 4:**
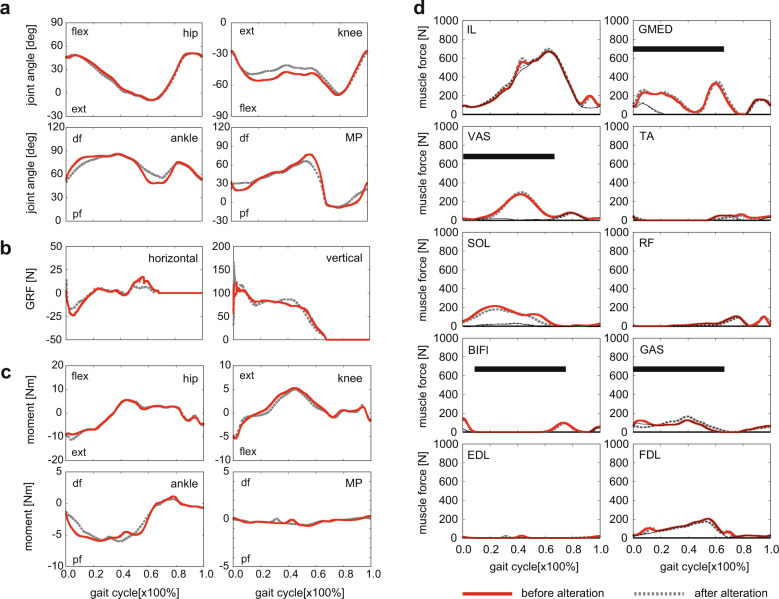
Comparisons of the changes in joint angles, ground reaction forces, joint moments, and muscle forces between before and after the alteration in foot morphology. Changes in (**a**) joint angles, (**b**) ground reaction forces (GRFs), (**c**) joint moments, and (**d**) muscle forces between before and after the alteration in foot morphology. Joint angles and moments were positive for hip flexion, knee extension, and ankle and metatarsophalangeal dorsiflexion. Horizontal GRFs were negative for breaking and positive for propelling components. Red solid line = before alteration. Gray dotted line = after alteration. Thick black solid bars indicate EMG activity profiles of five muscles during macaque’s bipedal locomotion^63^. Thick lines represent muscle forces due to both active contraction and passive elastic force generation. Thin black lines represent muscle forces generated by the passive elastic elements. The thin lines were mostly overwrapped with the corresponding solid lines in the IL, GAS and FDL muscles, indicating that the muscle forces were generated almost passively in these muscles.

We firstly compared the simulation of walking in the original Japanese macaque model with the unaltered foot and experimentally obtained walking data for evaluation of the simulation. Stick diagrams of the simulated and experimentally obtained bipedal locomotion of the Japanese macaque are shown in Fig. 2. The cycle duration, stride length, and duty factor of the generated locomotion were 0.71 s, 0.72 m, and 0.67, respectively, whereas those measured were 0.75 s, 0.79 m, and 0.65, respectively. A comparison between the generated joint angle profiles and those actually measured^62^ is shown in Fig. 3. The simulated results generally agreed with the measured data. A comparison between the generated vertical and horizontal reaction force profiles and those actually measured^62^ is also shown in Fig. 3. Japanese macaques generated a single-peaked vertical GRF profile, with the peak shifted toward the early stance phase. Although the force profiles did not match each other exactly, the simulated results generally agreed with the measured data and captured the main features of the GRF profiles in the Japanese macaque, such as the single-peaked vertical GRF profile with a peak occurring in the early stance phase, the breaking peak magnitude being slightly larger than that for propelling, and the breaking period being shorter than the propelling period^31^. The muscle force profiles (Fig. 4d) were also compared in an electromyographic (EMG) study of five hindlimb muscles from ordinary (not highly trained) Japanese macaques during bipedal locomotion^63^. The simulated muscle activation patterns were also generally in agreement with the reported EMG activity, except for the biceps femoris. In the simulated macaque locomotion, the largest contribution of the passive elastic (PE) element was observed in the iliopsoas muscle. The macaque is inherently a quadrupedal animal, and hip flexor muscle (in this model, iliopsoas) restricts hip-joint extension. When the macaque walks bipedally, the hip joint must be more extended than usual and the PE element contributes significantly. The PE element was also largely contributed in the gastrocnemius and flexor digitrum longus muscles during dorsiflexion of the ankle and MP joints. The estimated gross mass-specific metabolic cost of transport was 14.5 J/(kg m), whereas that estimated based on measured CO_2_ production rates^29^ was ~15 J/(kg m)^5^, indicating that the cost of transport was also reasonably well estimated. Therefore, our simulation framework successfully reproduced the basic kinematic and dynamic features of bipedal locomotion in the Japanese macaque, even though modeling of the musculoskeletal system was confined two-dimensionally. This simulation can be used to investigate predictively the effects of alterations in foot morphology on the kinematics, dynamics, and energetics of bipedal locomotion in the Japanese macaque.

### Predictive simulation with alterations in foot morphology

**Fig. 5:**
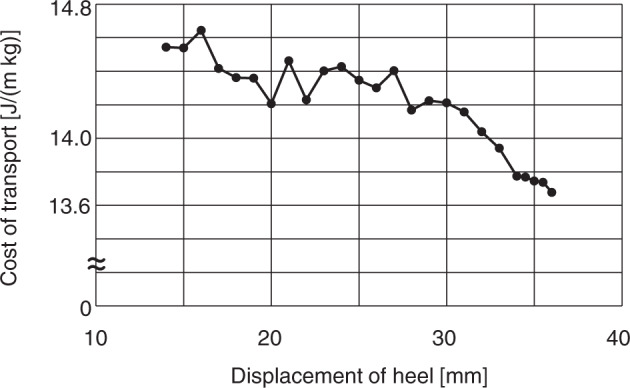
Change in the gross metabolic cost of transport as the heel was inferiorly translated. The change in the predicted gross metabolic cost of transport as the most posterior contact point of the foot corresponding to the calcaneal tuberosity was inferiorly translated is plotted. As the calcaneal point was inferiorly translated, the cost of transport gradually decreased compared with that of the original foot condition.

A stick diagram of the generated bipedal locomotion with altered foot morphology (the calcaneal tuberosity was inferiorly translated by 36 mm from the original position) compared with that of the intact foot is shown in Fig. 2. The cycle duration, stride length, and speed of the generated locomotion were changed from 0.71 s to 0.72 s, from 0.72 m to 0.70 m, and from 1.01 m/s to 0.97 m/s, respectively, indicating that the general spatiotemporal parameters of bipedal locomotion did not change as a result of the morphological alteration of the foot. A comparison of the joint angle, GRF, joint moment, and muscle force profiles of the simulated bipedal locomotion before and after the change in foot morphology is shown in Fig. 4 (the raw data are provided in Supplementary Data1). The joint angle profiles were generally similar between before and after the change in foot morphology, but the knee joint was more extended in the stance phase, and the ankle and MP joints were less plantarflexed and dorsiflexed, respectively, in the late stance phase after the foot morphology was altered (the magnitudes of the differences were ~5, 10 and 15 degrees, respectively). A notable difference was observed in the vertical GRF profile. Owing to the change in foot morphology, the vertical GRF profile shifted from a single- to a double-peaked profile as in human walking (the first and second peaks became ~15 N larger and the valley was reduced by ~10 N), although the second peak was not as prominent as that observed in human walking. The altered model showed a larger impact peak force at foot-strike than the original model, corresponding to the fact that the collision of the heel with the ground generates a large impact force, whereas the collision of the midfoot with the ground generates a smaller impact force in human walking^27^. In addition, the magnitudes of the breaking and propelling forces were substantially reduced if foot morphology was altered (the magnitudes of the reductions were ~5 N and 10 N, respectively). The joint moment and muscle force profiles were generally similar between before and after the foot alteration, but notable differences were observed in the ankle moment and soleus muscle force; the maximum force generated by the soleus and hence the ankle moment was decreased as a result of the altered foot morphology. The change in the predicted gross metabolic cost of transport as the most posterior contact point of the foot corresponding to the calcaneal tuberosity was inferiorly translated is shown in Fig. 5. As the calcaneal point was inferiorly translated, the cost of transport gradually decreased compared with that of the original foot condition. The % recovery, the rate of energy recovery via the pendular exchange of potential and kinetic energy^26^, of the bipedal locomotion generated before and after the alteration of foot morphology was 20.0% and 27.4%, respectively, indicating that the inverted pendulum mechanism was better utilized when foot morphology was altered.

## Discussion

If the foot of the Japanese macaque is changed to allow heel contact like a human foot, our simulation predicted that the knee was more extended in the stance phase, and that the ankle and MP joints were less plantarflexed and dorsiflexed, respectively, in the late stance phase. Kinematic comparisons of bipedal locomotion between Japanese macaques and humans^19^ have indicated that the ankle joint is more plantarflexed and that the foot segment is more vertically oriented at the time of toe-off in Japanese macaques than in humans. Therefore, the joint angles in the simulated bipedal locomotion were actually changed toward those observed in human and chimpanzee bipedal locomotion owing to altered foot morphology.

We also demonstrated that the metabolic cost of transport of bipedal locomotion decreased if foot morphology was altered. The proximate cause of this decrease is the reduction in the soleus muscle force (Fig. 4d). In the first half of the stance phase, the magnitude of the soleus muscle force was reduced by ~40 N because foot–ground contact with the heel was possible after the foot modification. As a consequence, the COP was shifted posteriorly and the GRF vector passed anterior but closer to the ankle joint, resulting in a reduction of the force necessary to be generated by the soleus to balance the moment exerted around the ankle joint in the dorsiflexing direction by the GRF. In the second half of the stance phase, the magnitude of the soleus force was reduced by ~70 N because a comparatively larger force was generated by the PE element of the gastrocnemius (~30 N larger) owing to the more extended knee and less plantarflexed ankle joints in the late stance phase. Consequently, the ankle joint moment decreased in the early and late stance phase in the altered foot model (Fig. 4c), and at that time, the ankle joint angle changed slowly (Fig. 4a), resulting in reduced muscle work of the ankle joint. Approximately 0.3 J larger elastic energy was stored in the PE element of the gastrocnemius at peak stretch in the altered foot model than in the original model. Therefore, because of the alteration of the foot, the elastic energy stored in the muscle spring was better utilized, and the metabolic cost of transport was reduced.

The change in foot morphology also evoked a change in GRF profile and whole-body mechanics during bipedal locomotion. Although the second peak was not as prominent as that observed in human walking, the double-peaked vertical GRF profile emerged in the altered foot model. The double-peaked vertical GRF profile is one of the most distinctive characteristics of human bipedal walking because nonhuman primates do not generally exhibit this force pattern^2^, and it is known to be functionally related to the efficiency of gait^64^. Because the force profile has two peaks in human walking, the net vertical force exerted to the body’s COM is greatest during the double-support phase and lowest during the mid-stance phase, which results in the vertical oscillation of the COM between a high point during the mid-stance phase and a low point during the double-support phase. Since horizontal velocity is lowest at the mid-stance phase and highest at the double-support phase, potential and kinetic energy fluctuate out of phase with almost equal amplitude in human bipedal walking like a simple pendulum. This out-of-phase fluctuation of the two types mechanical energy is the fundamental mechanism for saving energy in human walking^65^. The fact that the generated bipedal locomotion exhibited, although moderately, a double-peaked vertical GRF profile because of the alteration of foot morphology is therefore linked to the decrease in the cost of transport. Nevertheless, the vertical GRF of the altered foot model was not the clear two-peaked pattern of humans, but was more like that occasionally observed in chimpanzee bipedal walking^3^. Chimpanzees have a plantigrade foot and can make a heel contact during bipedal walking, but the heel and midfoot most often contact the ground simultaneously because the calcaneus is not as derived as that in humans. This fact possibly corroborates with the present result indicating the plantigrade foot leads to the emergence of the double-humped force curve in non-human primate bipedal locomotion.

The emergence of the double-peaked vertical GRF profile could be attributable to the fact that the forward translation of the COP started more posteriorly from the rear foot in the altered foot model. Because the GRF vector passed nearer to the ankle joint, the leg stiffness increased in the early stance phase in the altered foot model. The leg stiffness also increased in the late stance phase because the knee was more extended due to the fact that the whole-body kinematics and hence the COM movement was altered and the shank vaulted over the foot more slowly (Fig. 2d). A stiffer leg was accomplished in part through reduced plantarflexion at the ankle during push-off, which occurred because the PE element of the gastrocnemius is more stretched and hence larger ankle plantarflexion torque is generated to counterbalance the GRF acting to the plantar surface of the forefoot. Therefore, the slightly larger GRF was observed in the early and late stance phase, leading to the emergence of the double-peaked vertical GRF profile when the foot was altered to the human-like plantigrade foot allowing heel contact. In general, nonhuman primates possess more compliant legs than humans^66^. The peak vertical GRF profile decreases and the duration of the stance phase is prolonged if the leg is relatively compliant. This is the reason why nonhuman primates generally adopt so-called grounded running, defined as a gait utilizing spring-like running mechanics, even though the duty factor is >0.5 when moving on two legs^3–6,32^. In humans, however, grounded running is not generally observed. Humans utilize pendular mechanics if the duty factor is >0.5 and spring-like bounce mechanics if it is <0.5, and the transition from walking to running is discontinuous^67^. Therefore, the acquisition of walking gait utilizing the pendular mechanism by stiffening the stance leg is one of the critical transitions in the course of human evolution. The present predictive simulation implies that the evolution of the foot, particularly the acquisition of the human-like plantigrade foot possibly due to the evolutionary change of the calcaneus may facilitate generation of the two-peaked vertical GRF profile and improve the energetic efficiency of bipedal locomotion via the inverted pendulum mechanism, despite the fact that th`e other parts of the body are not yet specialized to the generation of bipedal locomotion. Improved locomotor economy is a strong evolutionary advantage for foraging and reproduction. The evolution of the foot could be a fundamental initial trigger for the evolution toward the acquisition of human-like bipedal locomotion. It must be noted that digitigrade terrestrial birds show a two-peaked GRF profile during bipedal walking^68–70^, and so do humans walking on toes^71^, indicating that plantigrade gait with human-like heel contact is not a necessary condition for the double-humped force curve. However, the present study indicated that the alteration of the foot from digitigrade to plantigrade foot could be one possible condition or scenario leading to the evolution of human-like bipedal walking for hypothetical hominids.

In human walking, the second force hump of the vertical GRF profile is generated due to activations of plantarflexor muscles such soleus and gastrocnemius, but in the macaque model, the forces generated by these triceps surae muscles is not as prominent as that in humans, resulting in the absence of the prominent second force hump. Therefore, in the macaque model, the second peak was generated in a different manner from human walking. However, this change observed in the macaque model also contributes to the energetic efficiency of bipedal gait, and the improved locomotor economy certainly is an evolutionary advantage.

A relatively human-like calcaneus with a large, robust tuberosity and plantarly located lateral plantar process^14,72,73^ accompanied by the fossilized footprints found in Laetoli^74,75^ suggest that *Australopithecus afarensis* walked with a prominent heel strike. Our simulation results imply that the vertical GRF profile of bipedal locomotion in *A. afarensis* exhibited the characteristic double-peaked vertical GRF profile, allowing utilization of the inverted pendulum mechanism at least higher than that observed in macaque bipedal locomotion with a single-peaked vertical GRF profile. The same is probably expected for *A. sediba*, because of its plantigrade foot, even though its calcaneus was relatively slender^76^. It must be noted, however, that although the bipedal locomotion generated in the present study was altered toward human-like bipedal locomotion, it is still essentially a bent-hip, bent-knee gait that is actually quite different from human bipedal locomotion, and the GRF profile is not the clear two-peaked pattern of humans. The %recovery of the generated bipedal locomotion is also relatively low (27.4%) and the value is within the range reported for chimpanzees (2–45%^7^). This fact could indicate that other anatomical constraints such as restricted lumbar spine mobility^77^, different gluteal morphology^78,79^ and/or restricted hip joint mobility owing to hip flexor muscles^31^ might also need to be modified for hypothetical protohominids to acquire human-like bipedal walking fully with improved utilization of the inverted pendulum mechanism. In addition, feet without a human-like longitudinal arch and prominent plantar aponeurosis^80–82^ should also be altered for effective propulsive force generation as observed in human walking. Further, the evolution of a larger body could be another possible prerequisite for the acquisition of bipedal walking with full utilization of the inverted pendulum mechanism because larger animals tend to take a more extended leg posture to decrease muscular forces by increasing muscle mechanical advantage^83^. The contributions of such anatomical factors to the acquisition of truly human-like bipedal locomotion should be investigated in future studies using the present predictive model.

One limitation of the present study is that the foot does not incorporate the so-called midtarsal break, a characteristic type of dorsiflexion at the midfoot during the stance phase observed across primate species^15,20,84,85^, including Japanese macaques^18,19^. However, the mobility of the midfoot is much lower than that of other joints, which suggests that the absence of the midfoot joint in our model had only a minor effect on the results of the present study. Another limitation was that we used simple 2D models of the musculoskeletal system and muscle force production. Major segmental movements during bipedal locomotion are confined to the sagittal plane, even in the Japanese macaque. Therefore, this was assumed to have only a minor impact on the predicted differences in the global body mechanics of bipedal locomotion. It must be noted, however, that omission of the force-length and force-velocity relationships of the muscles could have considerable effects on muscle gearing ratio changes^86^. In addition, although the muscle PE element was incorporated in the present study, no muscle series elastic elements, i.e., tendons, were included. As the simulated bipedal locomotion was not fast, the effect of the absence of tendons was thought to be small; however, the incorporation of a musculotendon model with correct identification of its mechanical parameters should also be investigated in future studies. Furthermore, the choice of 36 mm inferior translation of the calcaneal tuberosity as a human-like heel height was arbitrary and could have had important effects on the result of the current study. Lastly, although the simulated results generally agreed with the measured data and captured the main features of bipedal gait in the Japanese macaque, the GRF profiles did not closely match each other, possibly due to the problem associated with modeling of foot-ground contact mechanics, one of most challenging problems in the field of computational walking simulation^87^. For example, instantaneous vertical force development right after the foot-ground contact in the simulation (Fig. 4b) is one of the problems associated with the modeling. Further efforts are necessary to improve modeling of contact mechanics so that the simulated GRF closely resembles that of measured in future studies.

## Methods

### Musculoskeletal system model

**Fig. 6:**
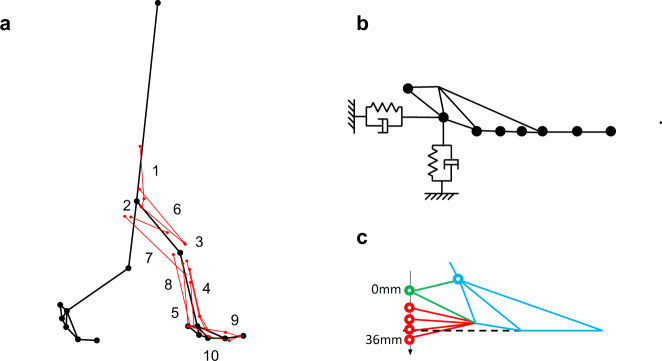
Two-dimensional musculoskeletal model of the Japanese macaque. **a** The model consists of nine rigid links and ten principal muscles. See Table 2 for the muscle numbers. Muscles were modeled as strings connecting the origin and insertion points via intermediary points. **b** Model of the contact between the foot and the floor. **c** Virtual alteration of foot morphology in the Japanese macaque. The heel of the foot corresponding to the calcaneal tuberosity was translated inferiorly by 1 mm up to 36 mm, so that it resembled that of humans.

We constructed a 2D musculoskeletal model of the bipedal Japanese macaque based on the anatomically based 3D whole-body musculoskeletal model^88^. The model consists of nine links representing the head, forelimbs, and trunk (HAT), thighs, shanks, and feet, which are represented by two parts—a tarsometatarsal and a phalangeal part—as shown in Fig. 6. The dimensions and inertial parameters of the limb segments were determined based on the 3D model (Table 1) as in Ogihara et al.^62^. Each joint was modeled as a hinge joint. The viscous property of each joint was modeled by a linear viscous element. The viscous coefficients of the hip, knee, and ankle joints were determined to be 0.109, 0.317, and 0.0943 Nms/rad, respectively, or one-tenth of the human values reported by Davy and Audu^89^ because the macaque was about one-tenth the mass of a human. The viscous coefficient of the MP joints was determined to be 0.01 Nms/rad, approximately one-tenth of that of the ankle joint. To prevent hyperextension of the knee joint, a nonlinear elastic element^89^ was attached:

1 $$T_{\mathrm{{knee}}} = - k_1\exp \left( { - k_2\left( {k_3 - \theta _{\mathrm{{knee}}}} \right)} \right)$$

where $T_{\mathrm{{knee}}}$ is the moment generated around the knee by the elastic element, $\theta _{\mathrm{{knee}}}$ is the knee joint angle, and *k*_1_, *k*_2_, and *k*_3_ are the coefficients determined as 1, 30, and −0.23, respectively.

**Table 1:** Dimensions and inertial parameters of the limb segments.

	Mass (kg)	Length (m)	COM (%)	M of I (kgm²)
HAT	8.184	0.482	52	2.07E−02
Thigh	0.557	0.163	41	1.61E−03
Shank	0.269	0.182	40	7.01E−04
Foot	0.080	0.074	62	5.49E−05
Phalanges	0.021	0.045	50	6.26E−06

Center of mass (COM) is represented as a fraction of segment length from the proximal end. M of I = moment of inertia about the COM. Parameters were taken from Ogihara et al.^62^, but the values were recalculated for the foot and phalangeal segments based on the CT-scanned surface data of the Japanese macaque^88^.

In this study, we considered ten muscle groups classified according to disposition (Fig. 6, Table 2). Each muscle was modeled as a string connecting the origin and insertion points^88^. In cases where the path of a muscle could not be described by a single line segment, an intermediary point was defined. These points were fixed to the corresponding bone coordinate systems. Thus, the moment arms of the muscles changed depending on the joint angles.

**Table 2:** Muscle parameters.

	Muscle group	F_max_ (*N*)	${\bar{\mathrm{L}}}$ (m)	Joint angles when the muscle length is ${\bar{\mathrm{L}}}$ (deg)			
1	IL	642^a^	0.153	H	80		
2	GMED	738^a^	0.086	H	34		
3	VAS	2514^a^	0.149	K	−45		
4	TA	390^a^	0.151	A	70		
5	SOL	822^a^	0.159	A	70		
6	RF	720^a^	0.189	H	25	K	−45
7	BIFl	804^a^	0.196	H	50	K	−45
8	GAS	720^a^	0.170	K	−65	A	60
9	EDL	140^b^	0.275	A	20	MP	0
10	FDL	180^b^	0.224	A	40	MP	0

*IL* iliopsoas, *GMED* gluteus medius + biceps femoris femoral part, *VAS* vastus, *TA* tibialis anterior, *SOL* soleus, *RF* rectus femoris, *BIFl* biceps femoris crural part, *GAS* gastrocnemius, *EDL* extensor digitorum longus, *FDL* flexor digitorum longus, *H* hip, *K* knee, *A* ankle*, MP* metatarsophalangeal joints.

^a^Values from Ogihara et al.^62^.

^b^Values estimated using the PCSA ratios (EDL or FDL): SOL based on Ogihara et al.^88^.

The force generated by a muscle was calculated based on the following equation:

2 $$f_m = F_m^{\mathrm{{MAX}}}a_m + f_m^{PE}$$

where *m* is the muscle number, $f_m$ is the muscular force generated by the *m*th muscle, $F_m^{\mathrm{{MAX}}}$ is the maximum force, $a_m$ is the activation signal, and $f_m^{PE}$ is the force generated by the PE element parallel to the contractile element. The maximum forces of the muscles were determined as in Ogihara et al.^62^ (Table 2). The force generated by the PE element was expressed by the following equation^45^:

3 $$f_m^{PE} = k_1^{PE}\left[ {\exp \left\{ {k_2^{PE}\left( {L_m - \bar L_m} \right)} \right\} - 1} \right]$$

where $L_m$ and $\bar L_m$ are the muscle length and the muscle rest length, respectively, and $k_1^{PE}$ and $k_2^{PE}$ are the coefficients. Therefore, the model accounted for elastic energy storage and release of the PE elements. The parameters $k_1^{PE}$and $k_2^{PE}$ were arbitrarily chosen to be 26 and 150, respectively, and then $\bar L_m$ was determined as shown in Table 2 by referring to the experimental data about the mobility of hindlimb joints in Japanese macaques^90^. The passive property of the muscle is actually different for each of the muscles, but since no data were available to determine each independent parameters, we assumed that $k_1^{PE}$and $k_2^{PE}$ were the same for all muscles but the passive property of the muscle could be reasonably well reproduced by adjusting the rest length of each muscle. To further reduce the number of parameters to be estimated in this model, the force-length and force-velocity relationships of the muscles were not considered.

Contact between the foot and floor was modeled by eight vertical and eight horizontal linear viscoelastic elements attached to the sole and phalangeal segment (Fig. 6). Based on Ogihara et al.^62^, the elastic and viscous coefficients were determined to be 6000 N/m and 60 Ns/m, respectively, for the vertical elements and 1200 N/m and 13 Ns/m, respectively, for the horizontal elements so as to successfully generate continuous bipedal locomotion of the model. During the swing phase of bipedal locomotion in the Japanese macaque, the proximal and distal interphalangeal joints are flexed to avoid contact between the toes and the ground; however, the present model does not have interphalangeal joints. Therefore, we allowed penetration of the phalangeal contact points into the ground during the swing phase once the leg underwent toe-off.

### Nervous system model

**Fig. 7:**
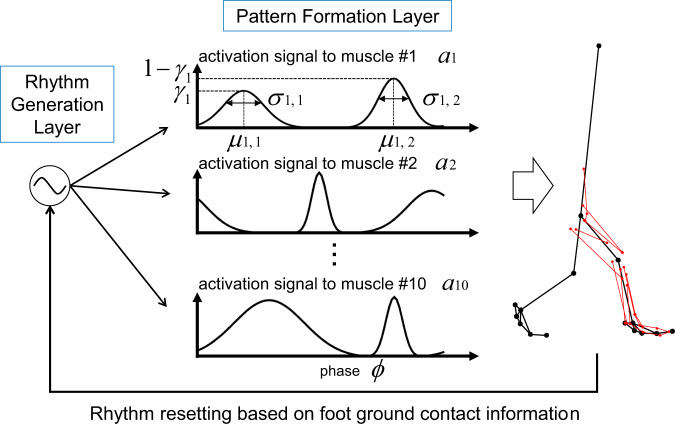
Mathematical model of the central pattern generator. The model consists of the rhythm generation layer modeled by a phase oscillator and the pattern formation layer representing muscle activation patterns by a combination of two Gaussian curves. $\bar a_m$ is the principal waveform of the *m*th muscle represented by the sum of two Gaussian functions (*m* = 1~10), $\gamma _m$ is the coefficient defining the relative contribution of the two bell-shaped curves (0 ≤ *γ*_*m*_ ≤ 1), $\mu _{1,m}$ and $\mu _{2,m}$ are the coefficients corresponding to the timing of the first and second peaks, respectively, and $\sigma _{1,m}$ and $\sigma _{2,m}$ are the coefficients corresponding to the width of the first and second bell curves, respectively. The phase of the oscillator $\phi$ was reset based on foot-ground contact events.

Animal locomotion, including that of primates, is generally accepted as being produced by a rhythm-generating neuronal network in the spinal cord known as the central pattern generator (CPG), with locomotion evoked by stimulus input from the mesencephalic locomotor region in the brain stem^91,92^. Such a spinal rhythm-generating neuronal network also seems to exist in primates and is hypothesized to contribute to the generation of actual locomotion^93,94^. Recent studies have suggested that the CPG consists of two layers: a rhythm generation (RG) layer that generates oscillatory signals and a pattern formation (PF) layer that generates muscle activity patterns based on the phase signal from the RG layer^95,96^. Therefore, in the present study, a mathematical model of the CPG consisting of the RG and PF layers was constructed (Fig. 7). The RG layer was modeled by two phase oscillators^49,50^ corresponding to the phase signals for the left and right legs as follows:

4 $$\dot \phi _R =	 \, \omega - K\sin \left( {\phi _R - \phi _L - \pi } \right)\\ \dot \phi _L =	 \, \omega - K\sin \left( {\phi _L - \phi _R - \pi } \right)\\ \omega = 	\, 2\pi /T$$

where $\phi _L$ and $\phi _R$ are the oscillatory phases of the left and right legs ($0 \le \phi _L,\phi _R \le 2\pi$), respectively, *ω* is the parameter defining the frequency of the oscillator (*2π*/*T*), *T* is the duration of the gait cycle, and *K* is a gain parameter. In this study, *T* and *K* were determined to be 0.747 s and 1, respectively, as in Ogihara et al.^62^. The PF layer then generated the activation pattern of each muscle represented by a combination of two Gaussian basis functions of the phase signal $\phi$ as follows:

5 $$\bar a_m(\phi ) = 	\, \frac{{\gamma _m}}{{\sqrt {2\pi } \sigma _{1,m}}}\exp \left( { - \frac{{\left( {\phi - \mu _{1,m}} \right)^2}}{{2\sigma _{1,m}}}} \right) + \frac{{(1 - \gamma _m)}}{{\sqrt {2\pi } \sigma _{2,m}}}\exp \left( { - \frac{{\left( {\phi - \mu _{2,m}} \right)^2}}{{2\sigma _{2,m}}}} \right)\\ a_m(\phi ) = 	\, \delta _m\frac{{\bar a_m(\phi )}}{{\max \bar a_m(\phi )}}$$

where $\bar a_m$ is the principal waveform of the *m*th muscle represented by the sum of two Gaussian functions, $\gamma _m$ is the coefficient defining the relative contribution of the two bell-shaped curves (0 ≤ *γ*_*m*_ ≤ 1), $\mu _{1,m}$ and $\mu _{2,m}$ are the coefficients corresponding to the timing of the first and second peaks, respectively, and $\sigma _{1,m}$ and $\sigma _{2,m}$ are the coefficients corresponding to the width of the first and second bell curves, respectively. This principal waveform was normalized by its maximum value and multiplied by the gain coefficient $\delta _m$ to generate the muscle activation. Therefore, a total of six parameters ($\gamma _m$, $\delta _m$, $\mu _{1,m}$, $\mu _{2,m}$, $\sigma _{1,m}$, and $\sigma _{2,m}$) were used to define the activation pattern of each muscle.

The RG layer in the CPG is known to modulate its basic rhythm by producing phase shifts and rhythm resetting based on sensory information^95,96^. To take this into account, we reset the oscillator phase $\phi$ based on foot–ground contact events^48,49^, since cutaneous afferents contribute strongly to phase resetting behaviors^97,98^.

In addition, we assumed a simple feedback control for postural control of the trunk segment by hip uniarticular muscles (the iliopsoas and gluteus medius) of the support leg as follows:

6 $$\begin{array}{l}a_{IL}^{pc} = \kappa \max (\bar \theta _{HAT} - \theta _{HAT},0)\\ a_{GL}^{pc} = \kappa \max (\theta _{HAT} - \bar \theta _{HAT},0)\end{array}$$

where $\theta _{HAT}$ is the trunk pitch angle with respect to the vertical axis, $\bar \theta _{HAT}$ is the reference angle of the trunk, and $\kappa$ is the gain parameter, determined empirically as 5.

### Generation of normal locomotion

To generate bipedal walking, an appropriate activation pattern must be determined for each of the ten muscles. In the present study, we used a genetic algorithm for tuning a total of 60 parameters defining the sequence of muscle activation patterns so as to minimize the following objective function *J*, given by the following:

7 $$J = \left\{ {\begin{array}{*{20}{c}} {1000J_{{\mathrm{distance}}}}& {{\mathrm{ if }}D\, \le\, 9m} \\ {w_1J_{{\mathrm{distance}}} + w_2J_{{\mathrm{cycle}}} + w_3J_{{\mathrm{HAT}}}} \\ { +\, w_4J_{{\mathrm{energy}}} + w_5J_{{\mathrm{joint}}} + w_6J_{{\mathrm{GRF}}}} & {{\mathrm{ if }}D\, > \, 9m} \end{array}} \right.$$

where $J_{{\mathrm{distance}}}$ is the distance term represented by the reciprocal of the distance traveled until the model falls down, *D*; $J_{{\mathrm{cycle}}}$ is the cycle term penalizing bipedal gait with the duty factor above or below the targeted duty factor (0.65^5^); $J_{{\mathrm{HAT}}}$ is the HAT term penalizing bipedal gait with a large HAT angle ($\theta _{HAT}$) fluctuation; $J_{{\mathrm{energy}}}$ is the energy term to minimize the gross metabolic cost of transport (mass-specific metabolic energy consumption per travelling distance) estimated based on the net positive and negative mechanical work done by the muscles and basal metabolic energy^62^; $J_{{\mathrm{joint}}}$ is the joint angle term representing the error in the joint angles between the generated and targeted (measured) bipedal gait at every foot–ground contact ($\phi = 0$); $J_{{\mathrm{GRF}}}$ is the GRF term penalizing bipedal gait with breaking forces applied to the phalangeal segment in the late stance and swing phases ($\pi \le \phi \le 2\pi$) to avoid tripping, and $w_{1 - 6}$ are the weighting coefficients. We used only the distance term in the early stage of the search process. The other terms were used later when the model could walk further than 9 m. We empirically determined $w_{1 - 6}$ to be 10, 1, 0.01, 0.1, 1, and 1, respectively, so that the energy term was weighted more than ten times more than the other terms because locomotion is basically determined to minimize the metabolic cost of transport^99,100^.

We constructed the musculoskeletal model of the Japanese macaque using the Open Dynamics Engine rigid body physics library (www.ode.org) with Microsoft Visual Studio 2010 C++, and then we solved the equation of motion using the implicit Euler method with a time step of 0.1 ms. We solved the differential equations of the nervous system using the Runge–Kutta method with the same time interval.

### Predictive simulation with alterations in foot morphology

To investigate how changes in foot morphology affect the kinematics, dynamics, and energetics of bipedal walking, we iteratively generated simulations for bipedal locomotion by changing foot morphology step by step. To achieve this, the most posterior contact point of the foot corresponding to the heel (calcaneal tuberosity) was translated inferiorly by 1 mm and the parameters were tuned to generate locomotion and minimize energy expenditure with the new foot morphology (Fig. 6c). This process was repeated until the overall foot morphology resembled the human plantigrade foot (36 mm below the original position, as this change in the vertical position of the heel in the macaque foot roughly corresponds to the human plantigrade foot morphology). Using our predictive simulation, we then evaluated how the kinematics, dynamics, and energetics of bipedal locomotion change as a result of alterations in foot morphology. The rate of energy recovery via pendular exchange of potential and kinetic energy (%recovery) was calculated as described in Ogihara et al.^5^.

### Reporting summary

Further information on research design is available in the Nature Research Reporting Summary linked to this article.

## Supplementary information

Description of Additional Supplementary Files

Supplementary Data 1

Reporting Summary

## Data Availability

The datasets generated during and/or analysed during the current study are available from the corresponding author on reasonable request.

## Code availability

The computer code used for this study is available from the corresponding author upon reasonable request.
