# Supplementary material for: Forward dynamic simulation of Japanese macaque bipedal locomotion demonstrates better energetic economy in a virtualised plantigrade posture
Source: Commun Biol. 2021 Mar 8;4:308. doi: 10.1038/s42003-021-01831-w (PMC7940622; doi:10.1038/s42003-021-01831-w)
Supplement: Supplementary file 1 — Description of Additional Supplementary Files [file 42003_2021_1831_MOESM1_ESM.pdf]

Description of Additional Supplementary Files

File name: Supplementary Data 1

Description: Comparisons of the changes in joint angles, ground reaction forces, joint moments, and muscle forces between before and after the alteration in foot morphology.
