# Supplementary material for: Forward dynamic simulation of Japanese macaque bipedal locomotion demonstrates better energetic economy in a virtualised plantigrade posture
Source: Commun Biol. 2021 Mar 8;4:308. doi: 10.1038/s42003-021-01831-w (PMC7940622; doi:10.1038/s42003-021-01831-w)
Supplement: Supplementary file 3 — Reporting Summary [file 42003_2021_1831_MOESM3_ESM.pdf]

nature research

Corresponding author(s): Naomichi Ogihara

Last updated by author(s): 2020/11/06

Reporting Summary

Nature Research wishes to improve the reproducibility of the work that we publish. This form provides structure for consistency and transparency in reporting. For further information on Nature Research policies, see our Editorial Policies and the Editorial Policy Checklist.

Statistics

For all statistical analyses, confirm that the following items are present in the figure legend, table legend, main text, or Methods section.

n/a	Confirmed	
☒	☐	The exact sample size (*n*) for each experimental group/condition, given as a discrete number and unit of measurement
☒	☐	A statement on whether measurements were taken from distinct samples or whether the same sample was measured repeatedly
☒	☐	The statistical test(s) used AND whether they are one- or two-sided<br>*Only common tests should be described solely by name; describe more complex techniques in the Methods section.*
☒	☐	A description of all covariates tested
☒	☐	A description of any assumptions or corrections, such as tests of normality and adjustment for multiple comparisons
☒	☐	A full description of the statistical parameters including central tendency (e.g. means) or other basic estimates (e.g. regression coefficient) AND variation (e.g. standard deviation) or associated estimates of uncertainty (e.g. confidence intervals)
☒	☐	For null hypothesis testing, the test statistic (e.g. *F*, *t*, *r*) with confidence intervals, effect sizes, degrees of freedom and *P* value noted<br>*Give P values as exact values whenever suitable.*
☒	☐	For Bayesian analysis, information on the choice of priors and Markov chain Monte Carlo settings
☒	☐	For hierarchical and complex designs, identification of the appropriate level for tests and full reporting of outcomes
☒	☐	Estimates of effect sizes (e.g. Cohen's *d*, Pearson's *r*), indicating how they were calculated

Our web collection on statistics for biologists contains articles on many of the points above.

Software and code

Policy information about availability of computer code

Data collection	List of software used in this study for simulation: Open Dynamic Engine (www.ode.org), Microsoft Visual Studio C++, Matlab. The computer code used for this study is available from the corresponding author upon reasonable request.
Data analysis	List of software used in this study for data analysis: Microsoft Excel, gnuplot(www.gnuplot.info).

For manuscripts utilizing custom algorithms or software that are central to the research but not yet described in published literature, software must be made available to editors and reviewers. We strongly encourage code deposition in a community repository (e.g. GitHub). See the Nature Research guidelines for submitting code & software for further information.

Data

Policy information about availability of data

All manuscripts must include a data availability statement. This statement should provide the following information, where applicable:

- Accession codes, unique identifiers, or web links for publicly available datasets
- A list of figures that have associated raw data
- A description of any restrictions on data availability

Data for Fig. 4 were provided in Supplementary Information. Other datasets generated during and/or analyzed during the current study are available from the corresponding author on reasonable request.

Field-specific reporting

Please select the one below that is the best fit for your research. If you are not sure, read the appropriate sections before making your selection.

☐ Life sciences ☐ Behavioural & social sciences ☒ Ecological, evolutionary & environmental sciences

For a reference copy of the document with all sections, see nature.com/documents/nr-reporting-summary-flat.pdf

Ecological, evolutionary & environmental sciences study design

All studies must disclose on these points even when the disclosure is negative.

Study description	Forward dynamic simulation of bipedal walking in the Japanese macaque to investigate how the morphology of the foot affects kinematics, mechanics and energetics of the gait.
Research sample	Comparative data of the macaque locomotion was taken from Ogihara et al. (2011). The model was constructed based on the data reported in Ogihara et al. (2009). No new data were obtained in the present study.
Sampling strategy	Not applicable
Data collection	Comparative data were taken from literatures.
Timing and spatial scale	Not applicable
Data exclusions	Not applicable
Reproducibility	Predictive simulation with alterations in foot morphology was conducted twice to confirm the reproducibility. Both generated essentially the same results.
Randomization	Not applicable
Blinding	Not applicable

Did the study involve field work? ☐ Yes ☒ No

Reporting for specific materials, systems and methods

We require information from authors about some types of materials, experimental systems and methods used in many studies. Here, indicate whether each material, system or method listed is relevant to your study. If you are not sure if a list item applies to your research, read the appropriate section before selecting a response.

Materials & experimental systems

n/a	Involved in the study	
☒	☐	Antibodies
☒	☐	Eukaryotic cell lines
☒	☐	Palaeontology and archaeology
☐	☐	Animals and other organisms
☒	☐	Human research participants
☒	☐	Clinical data
☒	☐	Dual use research of concern

Methods

n/a	Involved in the study	
☒	☐	ChIP-seq
☒	☐	Flow cytometry
☒	☐	MRI-based neuroimaging

Animals and other organisms

Policy information about studies involving animals; ARRIVE guidelines recommended for reporting animal research

Laboratory animals	This study used previously published data of macaque gait and anatomy. No new data were obtained in the present study.
Wild animals	This study did not involve wild animals.
Field-collected samples	This study did not involve field-collected samples.
Ethics oversight	The present simulation study does not require any ethical approvals.

Note that full information on the approval of the study protocol must also be provided in the manuscript.
